# Supplementary material for: A comparison of wild boar and domestic pig microbiota does not reveal a loss of microbial species but an increase in alpha diversity and opportunistic genera in domestic pigs
Source: Microbiol Spectr. 2024 Aug 20;12(10):e00843-24. doi: 10.1128/spectrum.00843-24 (PMC11448168; doi:10.1128/spectrum.00843-24)
Supplement: Supplemental figures and table — Fig. S1 to S6; Table S1. [file spectrum.00843-24-s0001.pdf]

Supplementary Table 1: The top 61 bacterial families (cut-off, 0.1% relative abundance in domestic and wild pigs) were observed in both groups. Families that were only present in the domestic pigs were labeled with the "#" sign.

Family	Mean Relative Abundance (%)	
	Domestic pigs	Wild pigs
f__Prevotellaceae	19.366	23.059
f__Lachnospiraceae	6.937	12.321
f__Oscillospiraceae	6.736	8.184
f__Rikenellaceae	6.690	9.231
f__Muribaculaceae	4.811	5.524
f__Spirochaetaceae	4.466	3.128
f__Lactobacillaceae	3.917	0.693
f__Ruminococcaceae	2.813	4.330
f__Christensenellaceae	2.505	0.684
f__Clostridiaceae	2.484	0.102
f__Bacteroidaceae	2.151	3.008
f__Selenomonadaceae	1.963	0.515
f__Bacteroidales_RF16_group	1.812	2.684
f__Peptostreptococcales-Tissierellales#	1.715	0.000
f__Streptococcaceae	1.563	0.052
f__Acidaminococcaceae	1.533	2.324
f__Succinivibrionaceae	1.486	0.607
f__Campylobacteraceae	1.448	0.108
o__Oscillospirales_f__UCG-010	1.354	1.173
f__Erysipelotrichaceae	1.346	0.579
f__Erysipelatoclostridiaceae	1.202	0.680
f__Porphyromonadaceae#	1.163	0.000
f__Peptostreptococcaceae	1.160	0.051
f__Clostridia_vadinBB60_group	1.144	0.849
f__Tannerellaceae	1.140	3.281
f__Veillonellaceae	1.098	0.620
f__Fusobacteriaceae#	1.080	0.000
f__F082	1.046	0.383
f__p-251-o5	0.996	3.020
f__[Eubacterium]_coprostanoligenes_group	0.965	0.706
f__Anaerovoracaceae	0.942	0.538
f__Enterobacteriaceae	0.818	0.034
f__Paludibacteraceae	0.772	0.278

f__Clostridia_UCG-014	0.713	0.405
f__Actinomycetaceae	0.562	0.002
f__p-2534-18B5_gut_group	0.556	1.649
f__WCHB1-41	0.425	0.551
f__Fibrobacteraceae	0.381	0.815
f__Aerococcaceae#	0.319	0.000
f__Moraxellaceae#	0.314	0.000
f__RF39	0.309	0.092
f__Butyricicoccaceae	0.283	0.329
f__Gastranaerophilales	0.270	0.725
f__Helicobacteraceae	0.262	0.010
f__Desulfovibrionaceae	0.259	0.358
f__Sutterellaceae	0.254	0.559
f__Planococcaceae#	0.251	0.000
f__Corynebacteriaceae#	0.211	0.000
o__Bacteroidales	0.184	0.779
f__Monoglobaceae	0.168	0.230
f__Mycoplasmataceae#	0.167	0.000
o__Rhodospirillales;f__uncultured	0.161	0.605
f__Puniceicoccaceae	0.144	1.292
f__Izemoplasmatales	0.142	0.028
f__Pasteurellaceae	0.141	0.044
f__Acholeplasmataceae	0.139	0.214
f__Bifidobacteriaceae	0.134	0.318
f__Carnobacteriaceae#	0.130	0.000
f__Pseudomonadaceae	0.124	0.003
f__Bradymonadales	0.117	0.130
f__Synergistaceae	0.113	0.053
Other	2.142	2.069

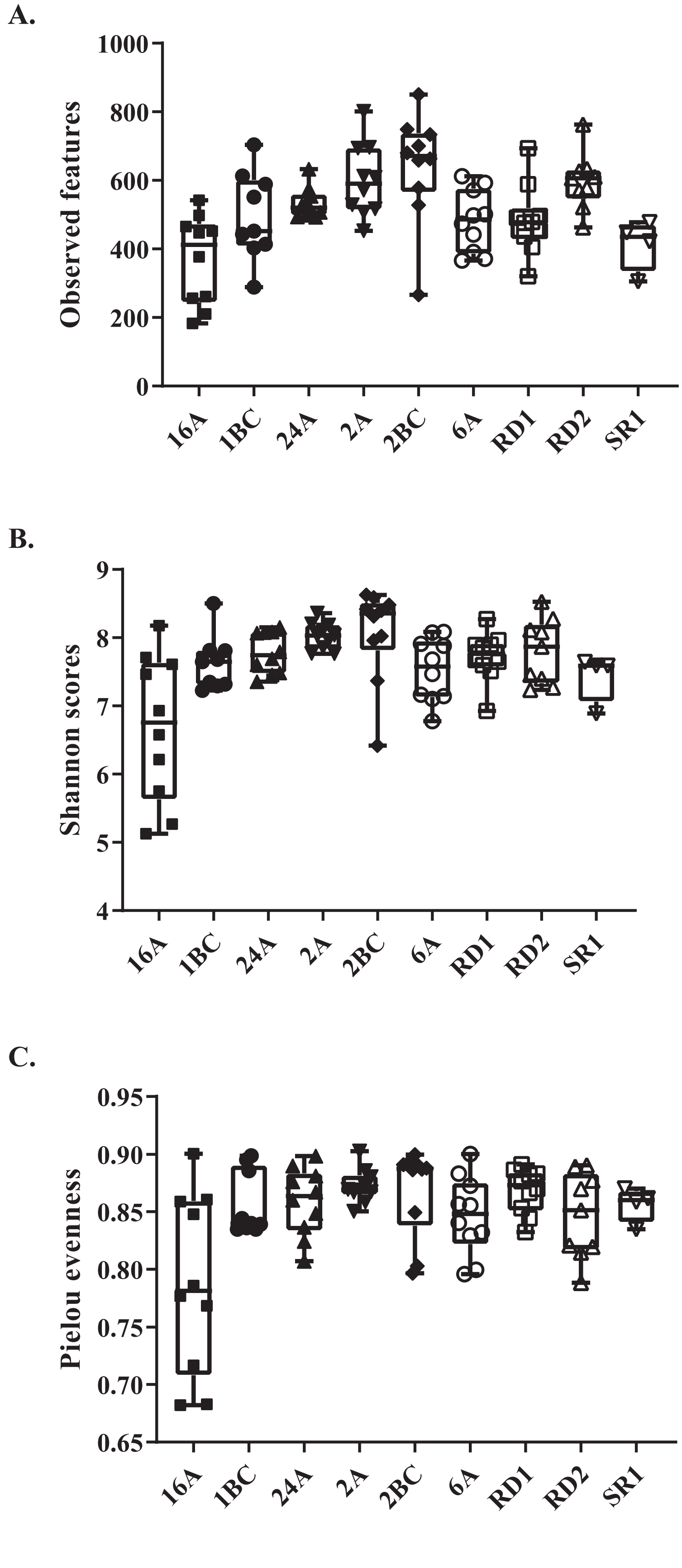

Supplementary Figure 1: Boxplot showing the comparisons of fecal microbial α-diversity indices among the 9 domestic farms using the Kruskal-Wallis test. (A) Based on the Observed features, the microbial richness analysis exhibited significant differences ($P < 0.0001$) across the farms. (B) Microbial diversity measured by Shannon index also showed significant variations ($P = 0.002$) among farms. (C) A trend in microbial evenness based on Pielou evenness scores among the domestic farms ($P = 0.07$). The boxes represent the interquartile range (IQR) between the first and third quartiles, with the black line inside each box indicating the median, and the whiskers extending to the lowest and highest values. The farms in Alberta include 16A, 24A, 2A, 6A, RD1, RD2, and SR1, while those in British Columbia are 1BC and 2BC.

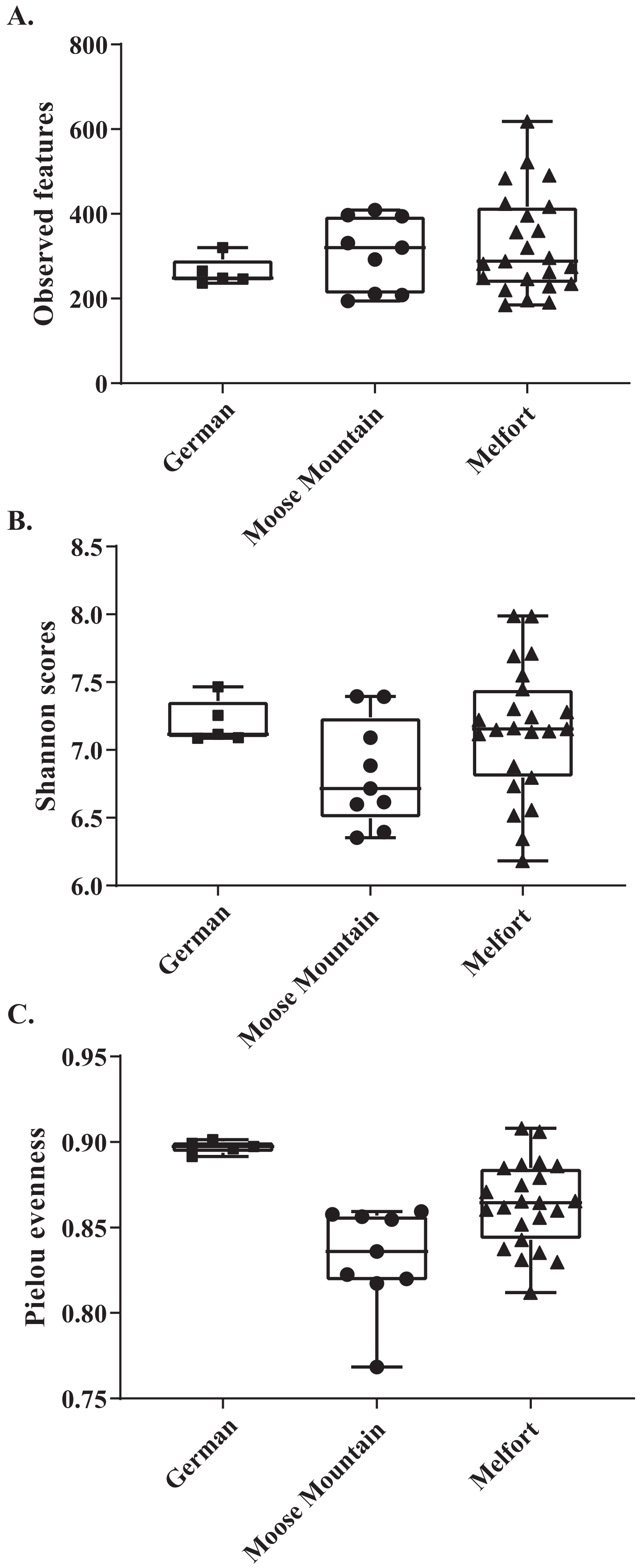

Supplementary Figure 2: Boxplot showing the comparisons of α-diversity indices of colonic microbial communities among the wild pigs from three locations using the Kruskal-Wallis test. No differences were observed for microbial richness ($P = 0.61$) based on the Observed features (A) and microbial diversity ($P = 0.17$) based on Shannon index (B). However, there were significant differences in the evenness ($P = 0.0004$) based on Pielou scores among the wild pigs' originating locations (C). The boxes represent the interquartile range (IQR) between the first and third quartiles, with the black line inside each box indicating the median, and the whiskers extending to the lowest and highest values.

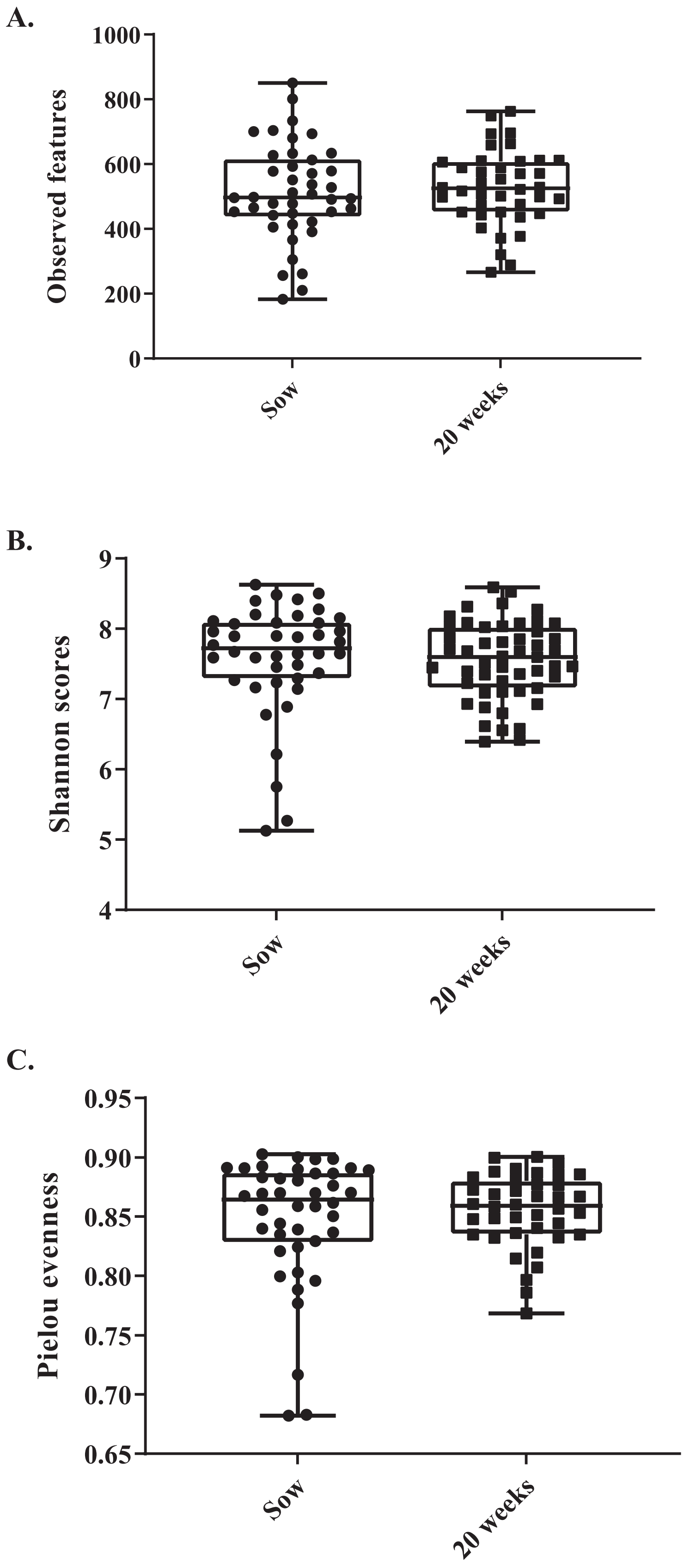

Supplementary Figure 3: Boxplot showing the comparisons of fecal microbial α-diversity indices between the Sow and 20-week groups of domestic pigs using the Mann-Whitney test. No differences were observed for microbial richness (*P* = 0.6) based on the Observed features (A), microbial diversity (*P* = 0.24) based on Shannon scores (B), or evenness (*P* = 0.76) based on Pielou evenness scores (C) between two age groups of the domestic pigs. The boxes represent the interquartile range (IQR) between the first and third quartiles, with the black line inside each box indicating the median, and the whiskers extending to the lowest and highest values.

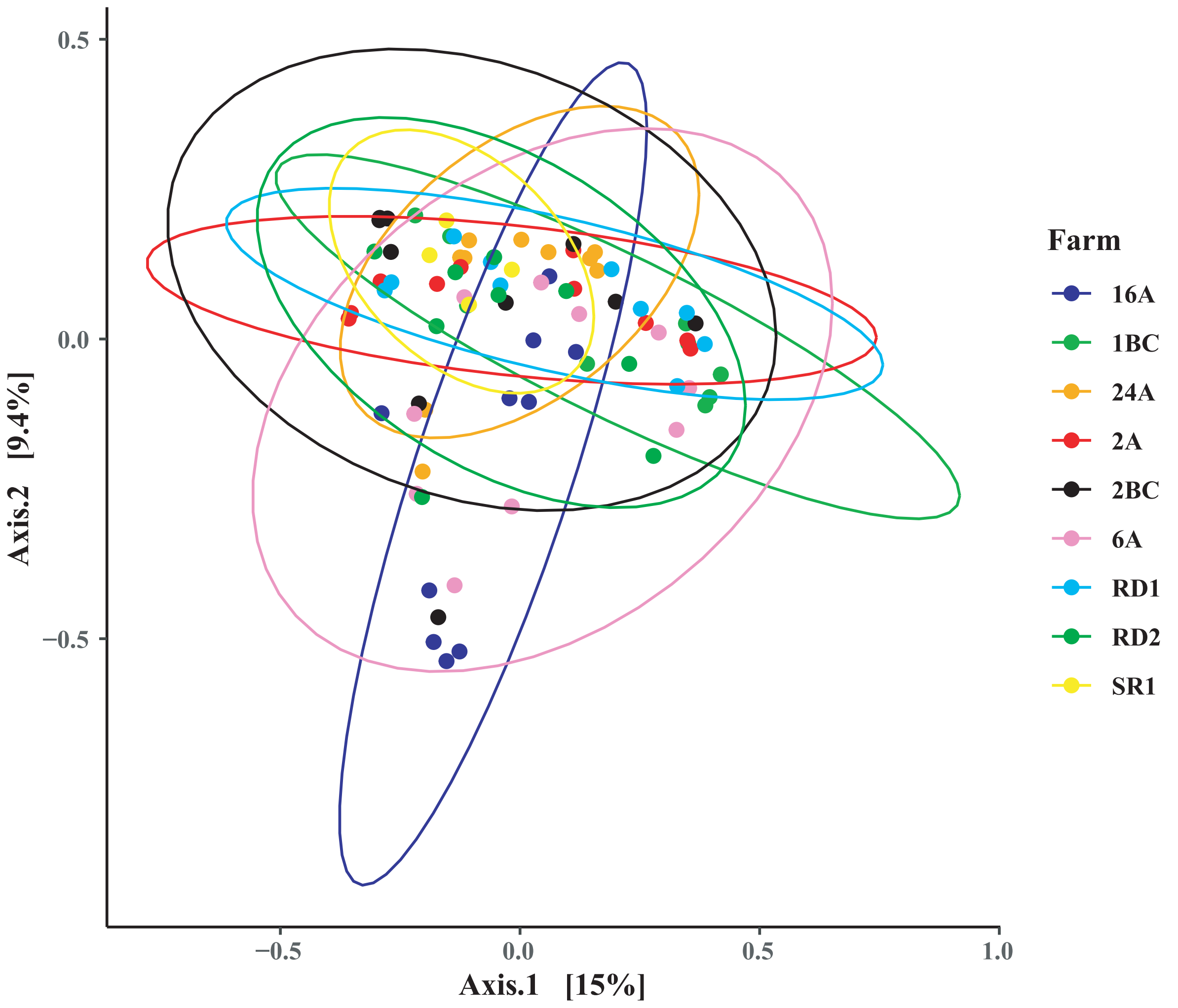

Supplementary Figure 4: The microbial community structure was different among the domestic pig farms (Adonis $P = 0.001$, $R^2 = 0.203$; beta-dispersion $P = 0.96$) as measured based on Bray–Curtis dissimilarity matrix. Axis 1 = principal coordinate 1 (PC1); Axis 2 = principal coordinate 2 (PC2). The farms in Alberta include 16A, 24A, 2A, 6A, RD1, RD2, and SR1, while those in British Columbia are 1BC and 2BC.

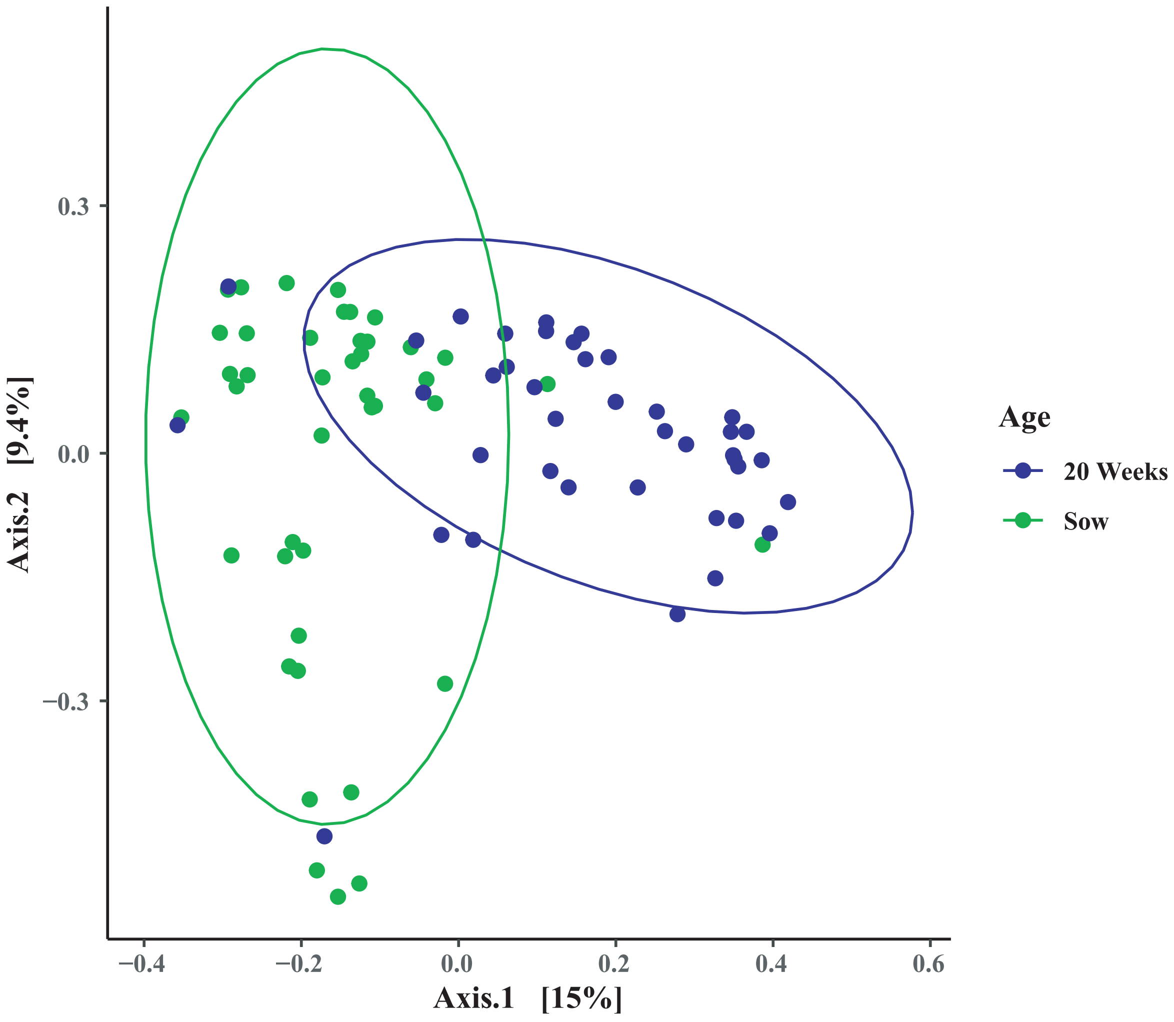

Supplementary Figure 5: The microbial community structure differed between the Sow and 20-week pigs (Adonis $P = 0.001$, $R^2 = 0.089$; beta-dispersion $P = 0.90$) as measured based on the Bray–Curtis dissimilarity matrix. Axis 1 = principal coordinate 1 (PC1); Axis 2 = principal coordinate 2 (PC2).

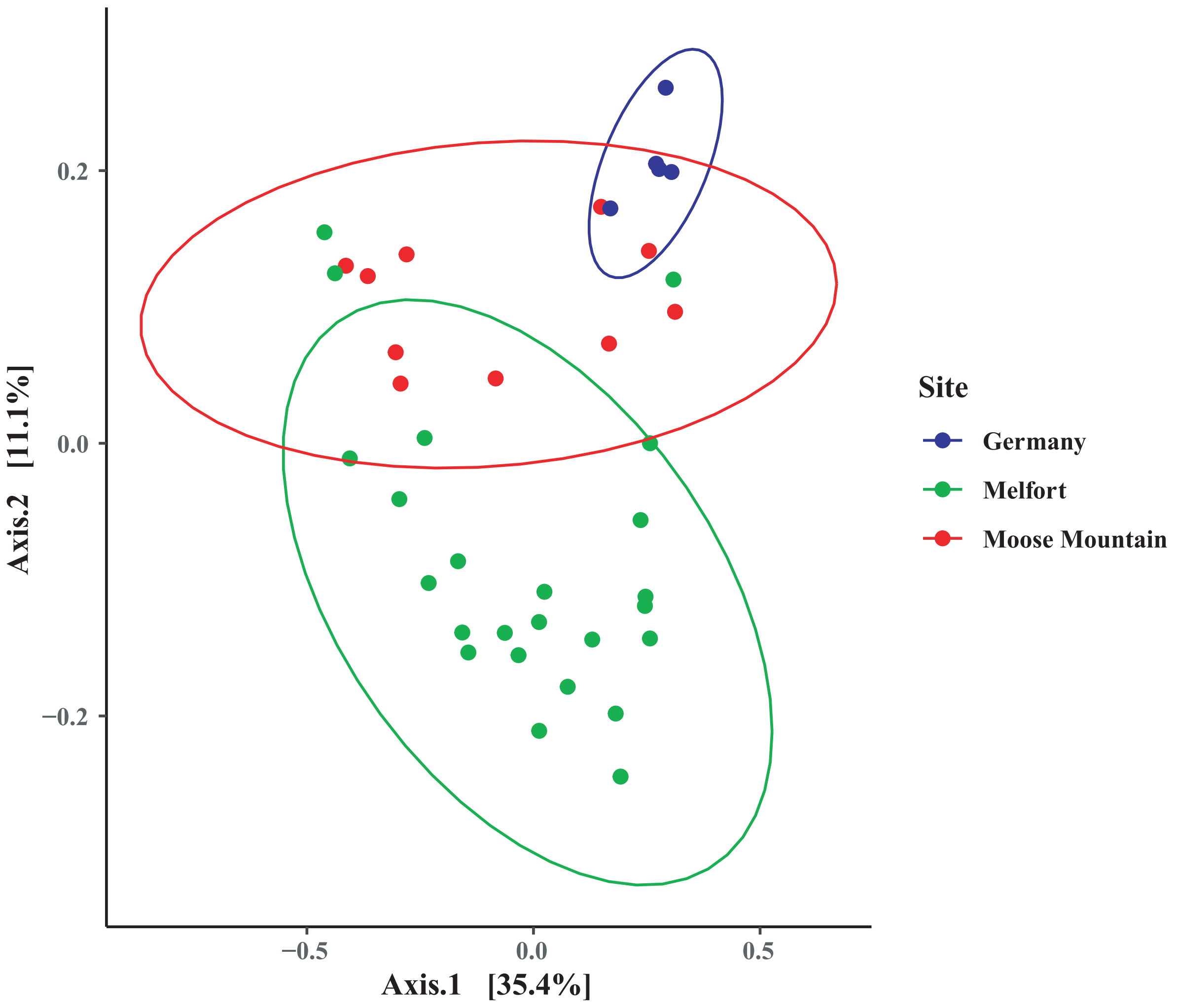

Supplementary Figure 6: The microbial community structure was different among the wild pig originating locations (Adonis $P = 0.001$, $R^2 = 0.257$; beta-dispersion $P = 0.31$) as measured based on Bray–Curtis dissimilarity matrix. Axis 1 = principal coordinate 1 (PC1); Axis 2 = principal coordinate 2 (PC2).
